# Exosome-related protein CRABP2 is upregulated in ovarian carcinoma and enhances cell proliferation

**DOI:** 10.1007/s12672-022-00492-3

**Published:** 2022-05-16

**Authors:** Ning Li, Guocui Lin, Ying Zhang, Qingyu Zhang, Haitao Zhang

**Affiliations:** 1 Department of Hematology, Affiliated Hospital of Guangdong Medical University, Zhanjiang, 524001 China; 2 Department of Biochemistry and Molecular Biology, Guangdong Medical University, Zhanjiang, 524023 China; 3 Department of Obstetrics and Gynecology, Affiliated Hospital of Guangdong Medical University, Zhanjiang, 524001 China; 4 The Marine Biomedical Research Institute, Guangdong Medical University, Zhanjiang, 524023 China

**Keywords:** Ovarian carcinoma, Exosome protein, Bioinformatics

## Abstract

Ovarian cancer is the most lethal cancer among women worldwide. Early diagnosis of ovarian cancer is a considerable challenge for gynecological oncologists. The exosome is a new emerging biomarker pool for cancer. Herein, we apply integrative transcriptome analysis to discover a new exosome biomarker for the diagnosis of ovarian carcinoma. We found 2316 differentially expressed genes (DEGs), among which were 431 DEGs that coded exosome proteins. We demonstrated three potential biomarkers—CRABP2, SPP1, and TNFAIP6, which are higher in ovarian cancer and associated with poor prognosis. According to receiver operating characteristic (ROC) curve analysis, CRABP2 performs better than the currently used biomarker CA125 in the diagnosis of ovarian cancer. CRABP2 level significantly elevates as the malignant property increases. We further confirmed that CRABP2 is upregulated in clinical tumor tissues of ovarian cancer patients as the malignant levels increase. CRABP2 can be detected and upregulated in the exosome in ovarian cancer patient serum samples rather than in healthy controls. GSEA revealed that high expression of CRABP2 was positively correlated with mitochondria oxidative phosphorylation pathway. At the same time, overexpression of CRABP2 can upregulate oxidative metabolism-related genes such as CYP4A11 and promote cell proliferation. Our findings based on CRABP2 could advance current practice in diagnosing and treating ovarian cancer.

**Supplementary information:**

The online version contains supplementary material available at 10.1007/s12672-022-00492-3.

## Introduction

Ovarian cancer is the most lethal cancer among women. High lethality results from late diagnosis of the disease, with > 70% of patients diagnosed at an advanced stage [1]. Unlike most solid cancers, ovarian cancer rarely metastasizes throughout the blood or lymph system but rather disseminates by the peritoneal cavity. Ovarian cancer progresses aggressively throughout the peritoneal cavity and distant organs [2]. It metastasizes through the peritoneal cavity, which makes it asymptomatic in the early stages [3]. CA125 antigen is the clinical diagnosis biomarker currently applied in the screening of ovarian cancer, but large randomized trials show that CA125 is not good enough yet for population-based ovarian cancer screening [4]. CA125 level increasing is not a specific event in ovarian cancer, as in some common conditions such as acute pancreatitis, pelvic inflammatory, and endometriosis, CA125 levels are also elevated. Moreover, not all ovarian cancer cases have a high CA125 level [5, 6]. The urgency of finding reliable biomarkers for women is apparent. A specific and sensitive biomarker utilization will advance the early diagnosis of ovarian cancer, which would be effective in ovarian cancer prevention and elimination.

Gene expression profiling is widely used to highlight the gene pattern deregulated in ovarian carcinoma. However, different studies on different sample sizes, races, control designs, and platforms have led to inconsistencies in the identified DEGs. The heterogeneity of the results makes clinicians less eager to use it. Therefore, an integrated analysis of multiple microarray studies is helpful to discover reliable DEGs and provide novel biomarkers for the diagnosis of ovarian carcinoma.

Biopsy is necessary for the accurate diagnosis of cancer, but it is not convenient and increases the risk of cancer metastasis [7]. The liquid sample, including blood, urine, and eye fluid, will be more convenient for the routine test than biopsy. Therefore, the upregulated protein that is released to the extracellular space or budding out as an exosome will be a good biomarker for the early diagnosis of ovarian cancer [8]. Exosomes are cell-derived vesicles that are present in many eukaryotic fluid samples, for example, blood, urine, cerebrospinal fluid, etc. Exosomes contain parental cell secreted RNA, proteins, lipids, and metabolites that are reflective of the cell type of origin [9]. Identification of a new biomarker that exists in the exosome will provide a new perspective for the diagnosis of ovarian cancer at the early stage of the malignant event. In this study, we integrated multiple research data and uncovered a new ovarian cancer-associated gene (OCAG). We further pick out the OCAG that may exist in the exosome and is correlated with the prognosis of ovarian carcinoma. Finally, we identify that CRABP2 is upregulated in ovarian cancer tissues and can be detected at a higher level in the exosome of patients, which might serve as a biomarker and surmount the obstacles in the early diagnosis and targeted therapy of ovarian carcinoma.

## Methods

### Dataset collection and data processing

Gene expression profiles of ovarian carcinoma were obtained from the GEO database (www.ncbi.nlm.nih.gov/geo). The following keywords were used: ‘Homo sapiens’ and ‘ovarian cancer’ or ‘ovarian carcinoma’. The DataSets containing gene expression profiles of ovarian cancer and healthy ovary epithelial tissues were included in the present study. Studies with a sample number < 3 in each group were excluded. Four datasets were adapted in this systematic analysis. The GEO IDs of these four datasets were GSE14001, GSE18520, GSE26712, and GSE27651, with a total of 299 samples (29 healthy, 270 cancer).

### Data normalization and meta-analysis

All the datasets’ origin expression data were downloaded, and the different groups were classified and unified in different datasets. The new classified datasets were uploaded into a network analysis online website tool (integrative meta-analysis of expression data; www.networkanalyst.ca). A global meta-analysis for identifying DEGs in ovarian cancer was conducted using the rank product algorithm (Rank Prod package in R statistical software; www.r-project.org) implemented in the web‑based tool Network Analyst and normalized using quantile normalization. The datasets were subsequently processed and annotated to adjust the data format and class labels to a consistent style. The random-effects model was used for integrity check by calculating the pooled effect size using Cochran’s Q tests.

### Functional enrichment analysis of DEGs and discovery of the target gene

The Database for Annotation, Visualization, and Integrated Discovery (DAVID; https://david.abcc.ncifcrf.gov/knowledgebase) is a comprehensive set of functional annotation tools. In the present study, gene ontology (GO) enrichment analysis (including biological process, cellular component, and molecular function categories) was performed in terms of cellular component analysis for DEGs using the DAVID tool. The genes that were enriched in the terms “exosome protein” were extracted out.

### Gene expression associated with cancer parameter and survival analysis

The potential gene expressions on survival expectation of patients were inquired in a transcriptomic microarray database including 3431 ovarian cancer samples (http://csibio.nus.edu.sg/CSIOVDB/CSIOVDB.html). In this database, the input gene symbol of interest will return the gene expression profile to the different subtypes of ovarian cancer it has a correlation with tumor characteristics such as tumor grade, tumor stage, and survival ratio of patients with a different expression level of the interested gene.

### Human samples

The research protocol was approved by the Guangdong Medical University Affiliated Hospital research ethics committee. Patients diagnosed with ovarian carcinoma between December 2020 and April 2021 were included in this study. Informed consent was obtained before the following experimental and clinical data analysis and integration. The tumor samples were fixed with formalin. Blood samples were contributed by the patients, and the serums of volunteers were collected through centrifugation. The exosome was extracted by Wayen exosome isolation kit (EIQ3-01001), and we strictly followed their instruction for extraction of the exosome.

### Cell lines and cell culture

The ovarian cancer cell OVCA429 cell and NM cell were kindly gifted by Prof. S.W. Taso (The University of Hong Kong). The cells were maintained in Dulbecco’s Modified Eagle’s Medium (DMEM) supplemented with 10% fetal bovine serum, 100 µg/ml of streptomycin, and 100 U/ml of penicillin (Gibco) at 37 °C in a 5% CO_2_ humidified cell incubator.

### Immunohistochemistry (IHC)

Streptavidin Peroxidase Immunohistochemistry was used for tissues IHC in this study. After this, tissue IHC staining kit was purchased from MaoKangBio (cat: MM1705). We strictly follow the protocol provided by this vendor. Briefly, after deparaffinization, epitope retrieval was conducted and the slices were then incubated in 3% H_2_O_2_ and 0.1% Tween 20 at room temperature to inactivate endogenous peroxidase activity. Then, the slices were blocked by 5% goat serum for 30 min. 100 µl/slice of the Primary antibody for CRABP2 (rabbit polyclonal, 10225-1-AP) was used at a dilution of 1:200, and isotype IgG was used for negative controls, overnight at 4 ℃. 50 µl/slice biotinylated secondary antibodies and 50 µl/slices Streptavidin-HRP were used. Freshly prepared DAB was used on 50 µl/slice for tissue staining for 2–5 min. The investigator who performed IHC (Guocui lin) was blind to sample clinical data. The grading criteria were as follows: negative 0, weak 1, moderate 2, and strong 3. IHC scores were calculated as the mean by two pathologists.

### Statistical analysis

All experiments were tested in triplicate at the least, and data were represented as Means ± SEM on the displayed figures. An unpaired, 2-tailed Student’s T-test was conducted to compare the statistical significance between control and tested groups. One-way ANOVA with Dunnett’s method was performed for comparisons of multiple groups. The log-rank test was used for comparison of survival outcomes with Kaplan–Meier method. P values less than 0.05 were considered to be significant difference, *P < 0.05, **P < 0.01, ***P < 0.001, ****P < 0.0001. All the statistical analysis was performed with GraphPad 7.0.

## Results

### Three potential genes for the diagnosis of ovarian cancer were identified by integrative analysis

**Fig. 1:**
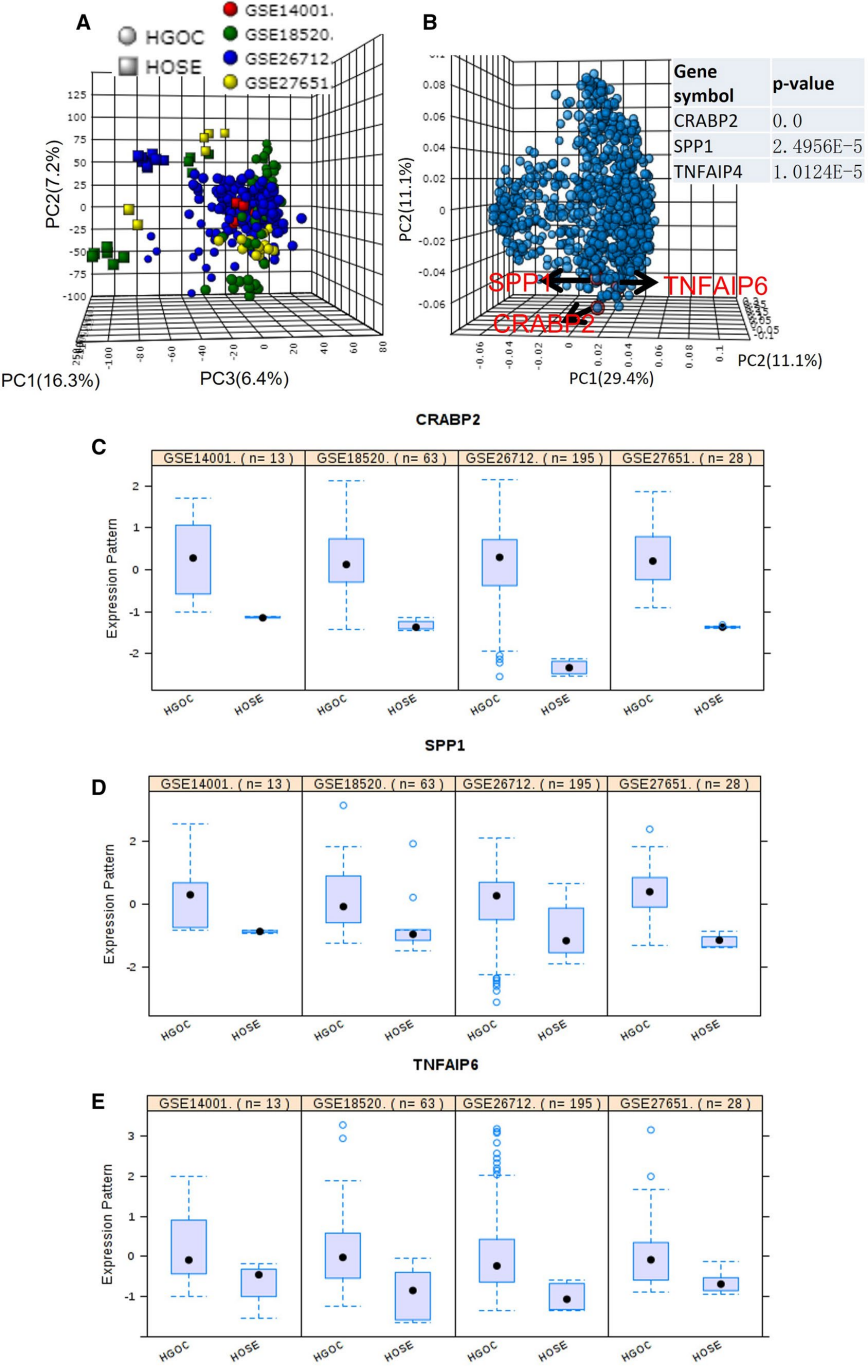
Identification of novel biomarkers of ovarian carcinoma using integrative analysis of multiple individual studies. **A** The 3D-PCA plot shows the distribution of all the samples based on transcriptional expression data. **B** The top 1000 differentially expressed genes’ distribution in a 3D-PCA plot is based on their expression pattern in all samples. **C** The expression of CRABP in all selected studies. **D** The expression of SPP1 in all selected studies. **E** The expression pattern of TNFAIP6 in all selected studies

Four individual studies’ data were integrated, and principal component analysis (PCA) was used to check the different sample qualities for the integrative analysis. The gene expression patterns between different groups (healthy vs. ovarian cancer) were significantly different according to the PCA plot (Fig. 1A). The differentially expressed genes were identified (Additional file 1), and these genes were presented into a 3D plot according to their expression pattern in different experiment groups (Fig. 1B). We performed GO analysis in terms of “cellular component”, and 431 genes were enriched in GO term “extracellular exosome”. Among these 431 genes, we found three genes (CRABP2, SPP1, and TNFAIP6) that coded exosome protein were significantly located at the bottom of this 3D plot, which indicates that these genes might be more significantly deregulated in ovarian cancer compared to other DEGs (Fig. 1B). The table on the right side of Fig. 1B indicated the combined ES effect of different studies and the P-value of these genes in the integrative analysis. We compared these genes expression patterns in the four datasets, and the results showed that all the three genes are dramatically upregulated in ovarian carcinoma in the four individual studies (Fig. 1C, E).

### CRABP2 is upregulated in ovarian carcinoma and is a prognostic marker

**Fig. 2:**
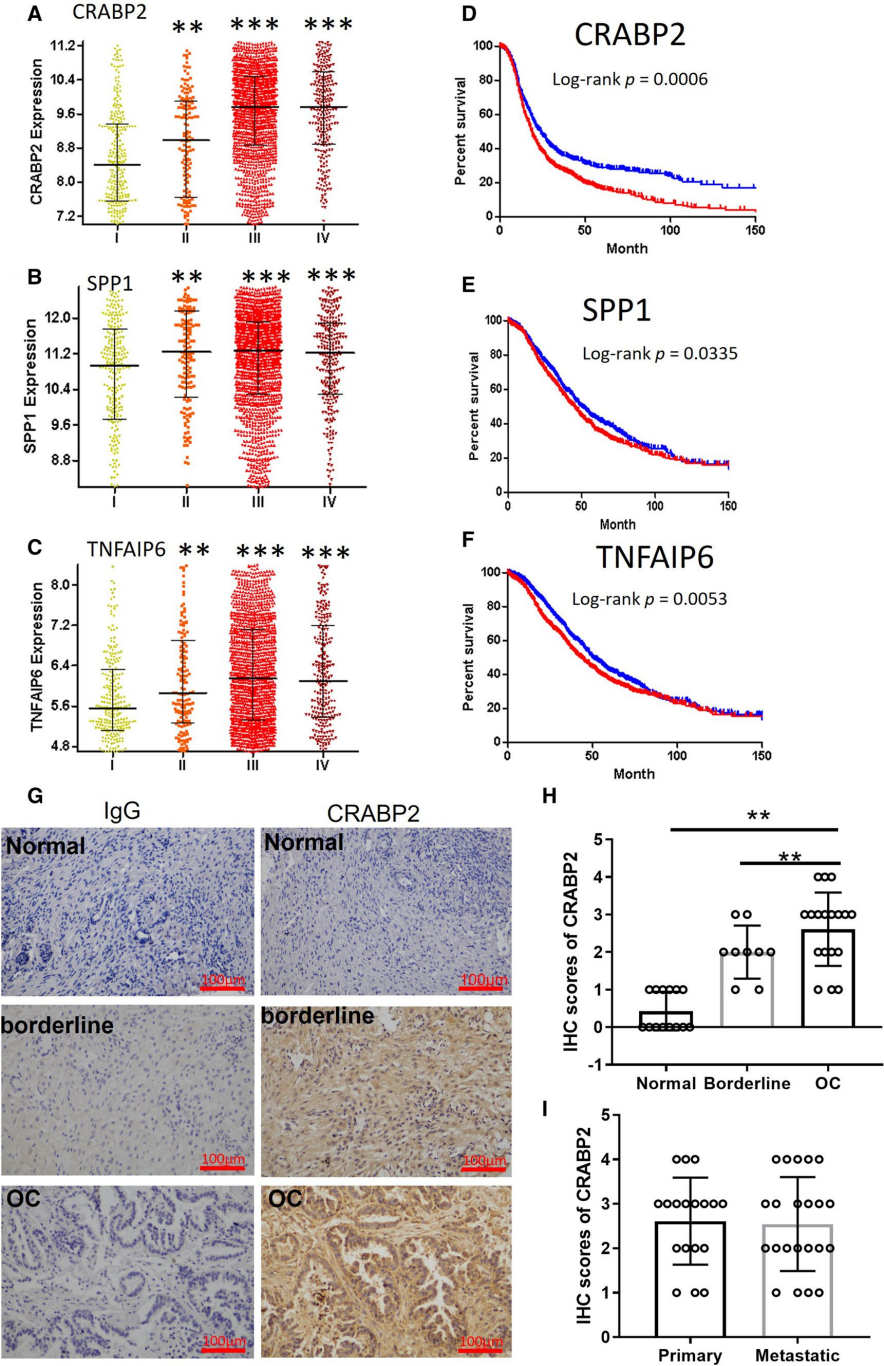
CRABP2 is upregulated in ovarian carcinoma and associated with cancer characteristic parameters. **A** CRABP2 expression in the different stages of ovarian cancer. **B** SPP1 expression in the different stages of ovarian cancer. **C** TNFAIP6 expression in the different stages of ovarian cancer. **D** The prognosis of CRABP2 was analyzed using the log-rank test. **E** The prognosis of SPP1 was analyzed using the log-rank test. **F** The prognosis of TNFAIP6 was analyzed using the log-rank test. **G** The presentative image of IHC staining of CRABP2 in ovarian cancer tumor tissues, borderline tumor, and normal tissue control. **H** IHC score statistical comparison of tissues sample in **G**. **I** IHC score statistical comparison of sample from the primary and metastatic tumor

Gene expression pattern disease is different with health control; DEGs only indicate the gene expression profile in ovarian cancer. In fact, not all DEGs participate in the disease’s progress, and some DEGs are the consequence instead of the cause of the disease. The oncogene functions on the progression and affects the prognosis of cancer. We want to discover a new biomarker that is not only a good indicator for the diagnosis of ovarian cancer but also a good prognosis marker for the evaluation of disease progression or regression. Therefore, we analyzed the gene expression at different stages of ovarian cancer. Our results claim that all three gene expression levels are increased as tumor stage (Fig. 2A–C). We also further analyzed the gene expression effect on the survival expectation of ovarian cancer patients. The result suggests these genes are a poor biomarker for the prognosis of ovarian cancer. These genes’ hazard ratio of high expression group (> median) compared to low expression group (< median) are as follows: CRABP2 [HR 0.84 (95% CI 0.7100–0.9108)] shorter than SPP1 [HR 0.8736 (95% CI 0.7713–0.9895)], and TNFAIP6 [HR 0.8375 (95% CI 0.7393–0.9487)] (Fig. 2D–F). To verify the CRABP2 expression in ovarian cancer, we collected ovarian cancer tissues from patients. Immunohistochemistry was applied to detect the protein content in tumor tissues, and the result indicated that CRABP2 level is higher in cancer tissues than their counterpart normal ovary tissues and borderline tumors (Fig. 2G, H). There is no difference in primary malignant tumor and metastatic tumor (Fig. 2I), which implies CRABP2 is majorly involved in cancer cell proliferation.

### CRABP2 promotes tumor growth and enhances cell oxidative phosphorylation

**Fig. 3:**
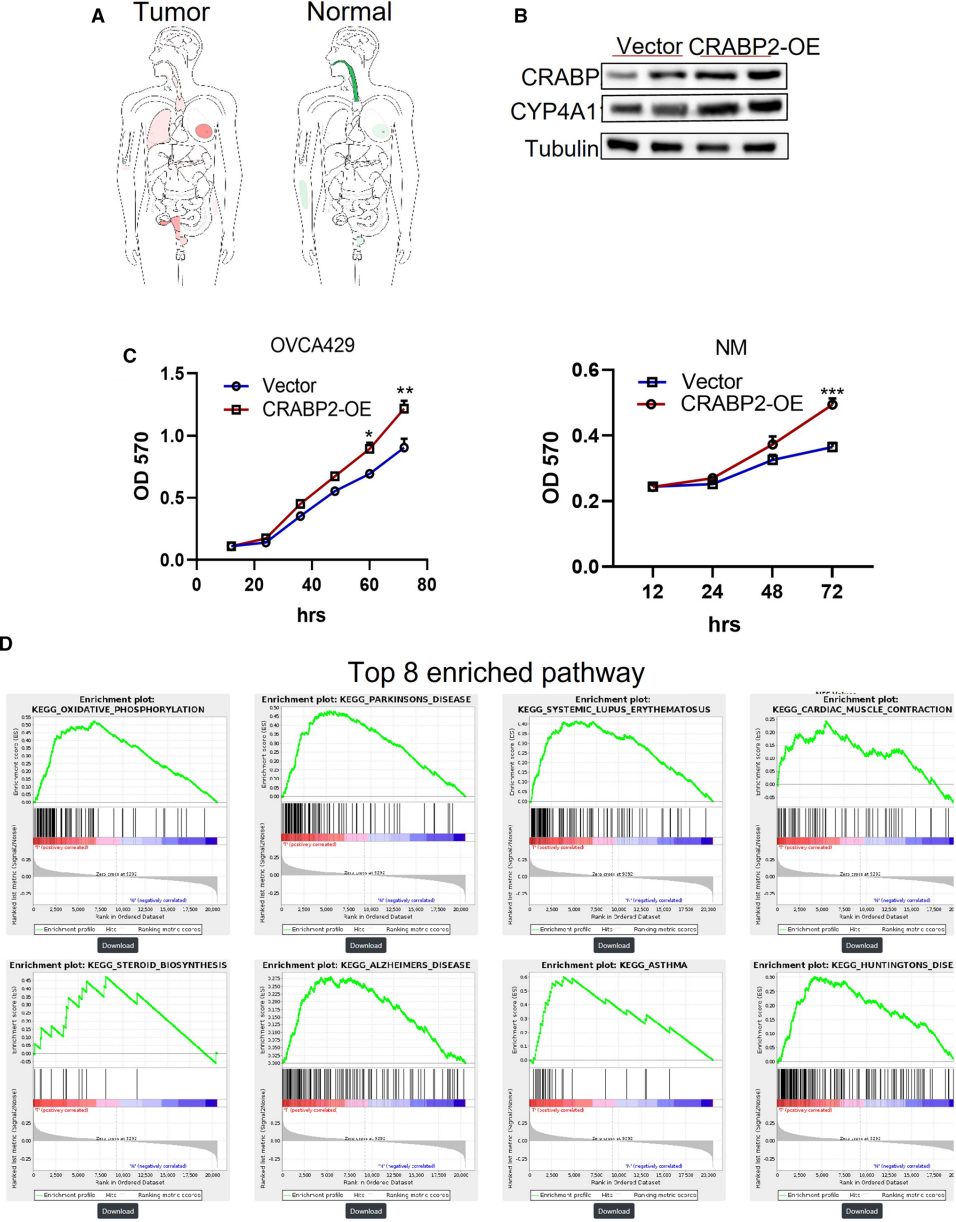
CRABP2 upregulation promotes cell oxidative phosphorylation and proliferation. **A** CRABP2 expression pattern in healthy tissues and tumor tissues of the whole-body organ. **B** Western blot was applied to detect CYP4A11 protein level after overexpression of CRABP2 in ovarian cancer cells. **C** CCK8 assay were performed to test cell proliferation after overexpression of CRABP2 p inovarian cancer cell line OVCA429 cells and NM cells. **D** GSEA of the pathway activated in CRABP2 upregulated ovarian cancer tumor tissues based on transcriptome data

In order to explore whether the expression of CRABP2 is a potential tumor-related gene, we first analyzed the changes in the expression of CRABP2 in all tumors through the GEPIA database (http://gepia2.cancer-pku.cn/#general), and the results showed that the expression of CRABP2 in ovarian cancer, lung cancer, endometrial cancer, and breast cancer was significantly increased (Fig. 3A), which implies that CRABP2 is an essential oncogenic gene in cancer. To clarify the role and mechanism of CRABP2 in promoting tumor progression, we overexpressed CRABP2 in ovarian cancer OVCA429 cells and NM cells, and the results showed that overexpression of CRABP2 promoted cell growth (Fig. 3C). We also performed GSEA pathway enrichment analysis on CRABP2 high expression versus CRABP2 low expression patient extracted from TCGA database (https://cgpe.soic.iupui.edu/gsea_pub_data/?, n = 308). The oxidative phosphorylation pathway gene expression was significantly increased (Fig. 3D), indicating that CRABP2 is involved in the regulation of oxidative phosphorylation, and overexpression of CRABP2 can induce the expression of fatty acid oxidative metabolism-related genes such as CYP4A11 (Fig. 3B), suggesting that CYP4A11 may mediate CRABP2 to promote cancer cell oxidation phosphorylation.

### CRABP2 expression level could be a potential diagnostic marker for ovarian cancer in exosomes

**Fig. 4:**
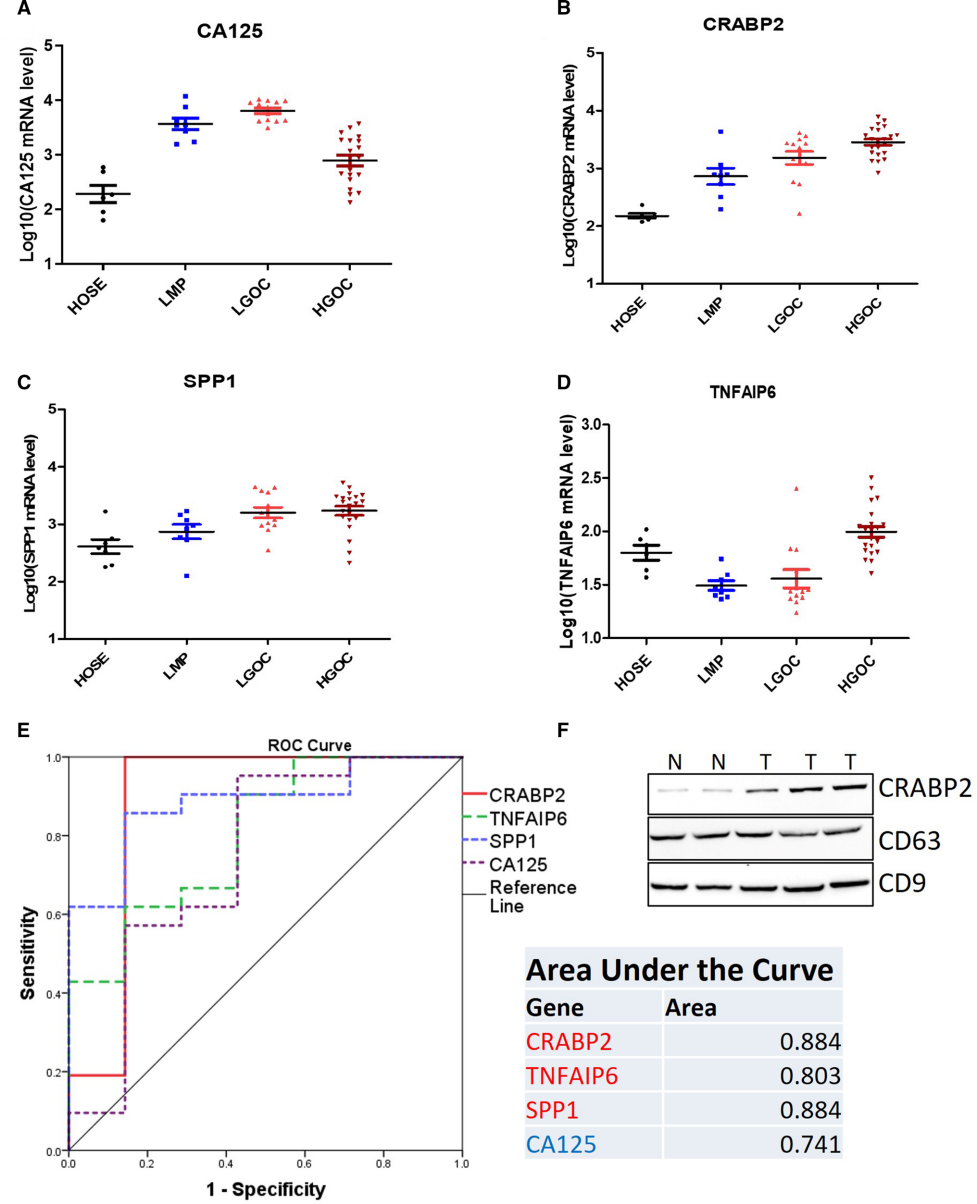
CRABP2 owns promising potential in the diagnosis of ovarian cancer. **A** The expression pattern of CA125 in HOSE, LMP, LGSOC, and HGSOC. **B** The expression pattern of CRABP2 in HOSE (n = 6), LMP (n = 8), LGSOC (n = 13), and HGSOC (n = 30). **C** The expression pattern of SPP1 in HOSE, LMP, LGSOC, and HGSOC. **D** The expression pattern of TNFAIP6 in HOSE, LMP, LGSOC, and HGSOC. **E** The diagnostic capability of the candidate gene and CA125 was evaluated by the ROC curve. **F** The CRABP2,CD63 and CD9 protein in serum exosomes were detected by immunoblot

As most of the ovarian cancer cases are diagnosed at an advanced stage, the mortality rates for ovarian cancer are the highest in women. CA125 is the biomarker used for screening ovarian cancer, especially in the BRCA1/2 mutation case. However, the CA125 level increased only in half of the early-stage ovarian cancer cases, and CA125 is also reported to increase in benign cysts such as ovarian endometrioma [10]. Our result also showed that the CA125 expression was significantly increased in low-grade serous ovarian carcinoma (LGSOC) and low malignant potential (LMP) ovarian tumors compared to human ovarian surface epithelial (HOSE) and even higher than its expression in high-grade serous ovarian carcinoma (HGSOC) (Fig. 4A). These results suggest that CA125 is not a good biomarker in the separation of low malignant ovarian tumor and ovarian cancer. To evaluate these candidate gene expressions in different degrees of malignant ovarian tumor, we compared these candidates’ expressions between LMP, LGSOC, and ovarian cancer. However, the three genes’ expressions, except TNFAIP6, are increasing as malignancy increase (Fig. 4B–D). To evaluate these gene capacities in the diagnosis of ovarian cancer, we further compared the CA125 level and the candidate genes expression level in the diagnosis of ovarian cancer using receiver operating characteristic (ROC) analysis. The results showed the three candidates performed better in the diagnosis of ovarian cancer than CA125 (Area under curve, AUC = 0.741). The AUC of CRABP2 and SPP1 even reached up to 0.884 (Fig. 4E). To answer whether the CRAPB2 exists in serum exosomes, exosomes were extracted and the CRABP2 was detected. CD63 and CD9 were used as biomarkers of exosomes. We demonstrated CRABP2 is existed in serum exosomes and is higher in patient samples than the counterpart of healthy control (Fig. 4F).

## Discussion

Ovarian cancer is the most lethal cancer in women, which is a big burden for society. The reason why ovarian cancer causes high mortality is that early diagnosis is almost unachievable. Because early ovarian cancer shows no obvious symptoms until the it metastasizes to the peritoneum and other organs, it results in floating and ascites accumulation. Transvaginal ultrasound (TVS) and the CA125 blood test are tests used most often to screen for ovarian cancer. TVS can help to find masses, but most of the masses are usually found to be not cancer. CA125 is used to help the clinician judge the tumor and drug treatment response because CA125 generally increases in most ovarian cancer cases, and the CA125 level will decrease when patients respond well to treatment [11]. But CA125’s low specificity and sensitivity makes it only a weak reference for clinical decision. In this study, we devoted ourselves to finding novel biomarkers with high sensitivity and specificity for the diagnosis of ovarian cancer.

Many studies have collected ovarian cancer samples, and the expression data are valuable in the GEO database. Here, we integrate the data of multiple studies and discover the significantly altered gene among all selected studies. The meta-analysis overcame the heterogeneity of individual studies and probably identify a novel gene that might serve as the diagnostic marker of ovarian cancer. In our study, we identify three exosome proteins which are CRABP2, TNFAIP6, and SPP1. Furthermore, these entire genes’ expression patterns are not only related to the tumor stage but also associated with the survival expectation of patients. SPP1 is reportedly involved in promoting ovarian cancer cell survival and progression [12, 13] and could be a good diagnostic marker for ovarian carcinoma [14]. Our result agrees with the previous conclusion that SPP1 functions as an oncogenic gene and could help in the detection of early ovarian cancer solely or in combination with CA125. The other two genes, CRABP2 and TNFAIP6, have not been reported as biomarkers of ovarian cancer. Here we found that TNFAIP6 expression is not positively related to the malignant tumor grade. Therefore, TNFAIP6 is weaker in the practice of diagnosis of ovarian cancer. But we found thatCRABP2 and SPP1 expressions increase as the malignant tumor property increases. CA125 expression in HGSOC is even lower than that in LMP ovarian tumors and LGSOC, which may answer why some tumor patients with high CA125 expression is not ovarian cancer [15], however the high-grade serous ovarian cancer CA125 is lower [16, 17]. Of note, CRABP2 expression is especially higher in ovarian cancer compared to the other two genes and has a closer relationship with the survival rate of patients. CRABP2 could be better in diagnosing ovarian cancer and even separating ovarian cancer and lower malignant tumors such as LMP ovarian tumors and LGSOC.

One of the main important problems regarding ovarian cancers is the recurrence after surgery and first-line chemotherapy. Usually, the recurrence of the disease poorly responds to first, and sometimes even second-line chemotherapy. In this scenario, it is possible that ovarian cancer’s inherent resistance may be due to reduced immunosurveillance and drug-resistant cells [14]. Metabolic reprogramming in ovarian cancer progression has been well-recognized by recent studies [17, 18]. Our study implies CRABP2 affects the mitochondria oxidation phosphorylation. It is possible that CRABP2 as an exosome protein could affect the neighbor cell and microenvironment which resulted in escaping of immunosurveillance and drug resistance by reprogramming cell metabolism. In conclusion, we integrated multiple studies and reduced the heterogeneity of individual studies and found easily-detectable exosome proteins, which might serve as new biomarkers for ovarian cancer diagnosis. We confirmed that the expression of these genes is related to tumor parameters and survival expectation. These results suggest that these genes may function as key proteins in the progression of ovarian cancer. We evaluated these gene expression levels and patterns in different degrees of malignant ovarian tumors. Finally, we concluded that CRABP2 is probably the best biomarker among these candidates identified by our approach. CRABP2 could be a promising ovarian cancer diagnostic and prognostic marker.

## Supplementary Information

**Additional file 1. **The differentially expressed genes were identified in three cohort studies by a meta-analysis.

## Data Availability

The authors confirm that the data supporting the findings of this study are available within the article.

## Abbreviations

DEGs: Differentially expressed genes
ROC: Receiver operating characteristic
AUC: Area under the curve
HOSE: Human ovarian surface epithelial
HGSOC: High-grade serous ovarian carcinoma
LGSOC: Low-grade serous ovarian carcinoma
PCA: Principal component analysis
OCAG: Ovarian cancer-associated gene
TVS: Transvaginal ultrasound
